# Long Lasting Cellular Immune Response Induced by mRNA Vaccination: Implication for Prevention Strategies

**DOI:** 10.3389/fimmu.2022.836495

**Published:** 2022-03-10

**Authors:** Laura Vitiello, Lucia Gatta, Sara Ilari, Stefano Bonassi, Mario Cristina, Filippo Ciatti, Massimo Fini, Stefania Proietti, Patrizia Russo, Carlo Tomino, Dolores Limongi

**Affiliations:** ^1^Laboratory of Flow Cytometry, IRCCS San Raffaele Roma, Rome, Italy; ^2^Scientific Direction, IRCCS San Raffaele Roma, Rome, Italy; ^3^Department of Health Science, Institute of Research for Food Safety and Health (IRC-FSH), University “Magna Graecia” of Catanzaro, Catanzaro, Italy; ^4^Clinical and Molecular Epidemiology, IRCCS San Raffaele Roma, Rome, Italy; ^5^Department of Human Sciences and Quality of Life Promotion, San Raffaele University, Rome, Italy; ^6^MEBIC, IRCCS San Raffaele Roma, Rome, Italy; ^7^Laboratory of Clinical Pathology, IRCCS San Raffaele Roma, Rome, Italy; ^8^Laboratory of Chronic and Neurodegenerative Diseases, IRCCS San Raffaele Roma, Rome, Italy

**Keywords:** SARS-CoV2, mRNA vaccination, T lymphocytes activation, B lymphocytes, COVID-19

## Abstract

As the COVID19 pandemic continues to spread and vaccinations are administered throughout the world at different rates and with different strategies, understanding the multiple aspects of the immune response to vaccinations is required to define more efficient vaccination strategies. To date, the duration of protection induced by COVID19 vaccines is still matter of debate. To assess whether 2-doses vaccination with BNT162b2 mRNA COVID-19 vaccine was sufficient to induce a persistent specific cellular immune response, we evaluated the presence of SARS-COV2 Spike-specific B and T lymphocytes in 28 healthcare workers 1 and 7 months after completing the vaccination cycle. The results showed that at 7 months after second dose a population of Spike-specific B lymphocytes was still present in 86% of the immunized subjects, with a higher frequency when compared to not-immunized controls (0.38% ± 0.07 vs 0.13% ± 0.03, p<0.001). Similarly, specific CD4+ and CD8+ T lymphocytes, able to respond *in vitro* to stimulation with Spike derived peptides, were found at 7 months. These results confirm that vaccination with BNT162b2 is able to induce a specific immune response, potentially long lasting, and could be helpful in defining future vaccination strategies.

## Introduction

As of December 3, 2021, more that 260 million cases of COVID-19 have been diagnosed, with more than 5 million deaths worldwide (1). Italy was the first European country to face the pandemic (2), and at the time of writing this manuscript, has counted more than 5 million confirmed cases and 134003 deaths (1). The introduction of mass vaccination changed the scenario (3), dramatically reducing the infection spread, and allowing governments to reduce or even dismiss restrictions that characterized the pre-vaccine management of the pandemic. To date, nearly 8 billion vaccine doses have been administered (1). However, the duration of protection induced by vaccination is still matter of debate. Data from patients infected in 2002 with SARS-COV indicates that post-infection T cell memory could last up to 11year (4), while the level of circulating antibodies rapidly decline below the detection limits (5). As regards SARS-COV-2 infection, evidence shows that neutralizing antibody titers against the SARS-CoV-2 spike protein persisted for at least 5 months after infection (6). Vaccination with mRNA that encodes a SARS-CoV-2 full-length spike protein induces seroconversion in the first month after the second dose, with neutralizing anti RBD (Receptor Binding Domain) IgG traceable up to 6 months (7). A decrease in spike-specific antibodies can be observed from the second month after vaccination (7) with lower concentration observed in males and older individuals (8). Decreased antibody levels, together with the insurgence of re-infections (9) - an event that suggest a waning of the immune response - prompted public discussion about vaccination strategies and the necessity of additional doses. However, immune response to viral infections rely also on the activation of a specific cellular response. Preliminary reports described the generation of a specific T- and B- cellular memory lasting up to 8 months after SARS-CoV2 infection (10). Here, we monitored the levels of anti-RBD IgG in a cohort of 432 Health Care Workers (HCW) at IRCCS San Raffaele Roma up to 7 months after completion of the vaccination cycle. In a subgroup of 28 subjects we investigated the persistence of Spike-specific T and B lymphocytes after 1- and 7-months post vaccination with the BNT162b2 mRNA vaccine. When we started the study (March 2021), little or no evidence was available on the persistence of cellular immune response after mRNA vaccination. So, the aim of our project was to evaluate whether cellular immune response to vaccination would be persistent in time.

## Materials and Method

### Study Participants

The study was approved by the Ethic Committee of IRCCS San Raffaele Roma (POST-VAX RP 21/07). The protocol has been registered on clinicaltrial.gov (NCT05102669). The study cohort for the detection of serum IgG was composed by 432 healthcare workers (250 females 57.8% of total; mean age 50, range 27-67; males 42.2% of the cohort n=182, mean age 51, range 31-72). For the analysis of specific cellular immune response, 28 vaccinated HCW and 25 not immunized subjects were enrolled. The group of immunized subjects was composed by 16 females and 12 males (mean age 48 years, range 26-77; female mean age 48 years, range 26-63; males mean age 47 years, range 27-77). The controls group of not immunized individuals was composed by 15 females (mean age 40 years, range 30-51) and 10 males (mean age 37 years, range 26-50). Mean age for control group was 39 years (range 26-51). All the subjects declared that they never tested positive for COVID19. The absence of asymptomatic infection was confirmed by the weekly nasal swabs and rapid antigenic planned by the hospital surveillance program for all workers (Tomino, C., et al. SARS-CoV-2 epidemiological surveillance of healthcare professionals working in an inpatient rehabilitation facility. submitted).

### Evaluation of Anti SARS-CoV-2 Antibodies

To evaluate total anti-SARS-CoV-2 antibodies, serum samples were collected from immunized subjects at months 1, 4 and 7 after completion of vaccination cycle. Anti-SARS-CoV-2 IgG ELISA was performed using SARS-CoV-2 IgG II Quant Reagent Kit (Abbott, Illinnois, USA). The measurement was performed by chemiluminescent microparticle immunoassay (CMIA) on a ARCHITECT analyzer (Abbott).

### Cell Isolation and Stimulation

For cellular immunity analysis, venous blood was obtained 1 and 7 months after the second dose for vaccinated subjects, while samples from control subjects were obtained once. All the subjects declared that they never tested positive for COVID19. Peripheral blood mononuclear cells (PBMCs) were isolated from whole blood by density gradient centrifugation according to manufacturer’s instructions (Pan Biotech). PBMC were washed and resuspended in freezing medium (FBS 10% DMSO) than stored at -80°C until the day of the assay. On the day before the assay, cells were thawed, resuspended in RPMI 1640 medium (Thermofisher) supplemented with 5% autologous serum, 100 U/ml penicillin (Thermofisher), 0.1 mg/ml streptomycin (Thermofisher). 1 ml of cell suspension (5x106cells/ml) was plated in 24-well plates and incubated at 37°C and 5%CO₂ overnight.

### Detection of Spike-Responding T Cells

On the day of stimulation PBMCs were harvested and counted. Cells were washed and resuspended in culture medium at a density of 1×10^7^ viable cells per mL. In brief, 100 µl of cell suspensions was seeded in flat-bottom 96-well plates and stimulated for 6 hours with or without PepTivator SARSCoV-2 protein S, S1 and S+ peptide pools (1 µg/ml each, Miltenyi Biotec, PepTivator SARS-CoV-2 Prot_S 130-126-700, cat # PepTivator SARS-CoV-2 Prot_S1, cat #130-127-041, and PepTivator SARS-CoV-2 Prot_S+, cat # 130-127-311). The PepTivator SARS-CoV-2 Prot_S contains the sequence domains aa 304-338, 421-475, 492-519, 683-707, 741-770, 785-802, and 885 – 1273 (sequence end); the PepTivator SARS-CoV-2 Prot_S1 contains the aa sequence 1–692 of the surface glycoprotein and Prot_S+ covers parts of the C-terminal S2 domain (aa 689–895)2 μL of CytoStim (Miltenyi Biotech), was used as positive control in a different well. After 2 hours of incubation, Brefeldin A (5µg/ml) was added to each well. After 4 hours, cells were collected for flow cytometry.

Cells were fixed and permeabilized then stained with CD3 Antibody, anti-human, APC,REAfinity™ (clone REA613, isotype: recombinant human IgG1); CD4 Antibody, anti-human, Vio^®^Bright B515, REAfinity (clone REA623, isotype: recombinant human IgG1) CD8 Antibody, anti-human, VioGreen™, REAfinity (clone REA734, isotype: recombinant human IgG1); IFN-γ Antibody, anti-human, PE, REAfinity (clone REA600, isotype: human IgG1); TNF-α Antibody, anti-human, PE-Vio^®^770, REAfinity (clone REA656, isotype: human IgG1); CD14 Antibody, anti-human, VioBlue^®^, REAfinity (clone REA599, isotype: human IgG1); CD20 Antibody, anti-human, VioBlue^®^, REAfinity (clone REA780, isotype: human IgG1); CD154 Antibody, anti-human, APCVio ^®^ 770, REAfinity (clone REA238, isotype: human IgG1) according to manufacturer instructions (Miltenyi Biotech SARS-CoV-2 Prot_S+ T Cell Analysis Kit (PBMC) human. Stained PBMC samples were acquired and analysed on FACS LSRFortessa (BD Biosciences), using FACSDiva software, v 8.0.2.

### Detection of Antigen-Specific B Cells

To detect SARS-CoV-2 specific B cells, isolated PBMC were incubated with recombinant biotinylated SARS-CoV-2 RBD (0,1µg). Cell were washed then incubated with streptavidin PE (BD) for 15’. After incubation cells were washed than stained with anti-CD45 BUV395, anti CD19 APC, anti CD27 APC-R700, anti IgD BV421 (all from BD Biosciences). Stained PBMC samples were acquired and analysed on FACS LSRFortessa (BD Biosciences), using FACSDiva software, v 8.0.2.

### Statistics

Sample size was predetermined using statistical methods. The experiments were not randomized and the investigators were not blinded to allocation during experiments and outcome assessment. Qualitative variables were presented as frequencies and percentages, quantitative variables were expressed as mean values and standard error (SE). Non-parametric statistical tests were used in case of non-normality: the normality of continuous variables was calculated by Kolmogorov-Smirnov test. Mann-Whitney U-test was used to compare the differences between not-immunized and immunized; while Wilcoxon signed-rank test was used to compare the differences between paired comparisons in the immunized between T1 and T7. Association between categorical variables was assessed by Chi-Square Test.

Differences in IgG in the subsets were analyzed with Friedman test and Dunn’s multiple comparison test; while the differences among IgG values in the main study were analyzed by repeated-measurement two-way ANOVA and Bonferroni’s multiple comparison test. A two-sided p < 0.05 was considered statistically significant. All analysis data were analyzed using SPSS Statistics (Version 27) and GraphPad Prisma (version 8.0).

## Results

**Figure 1:**
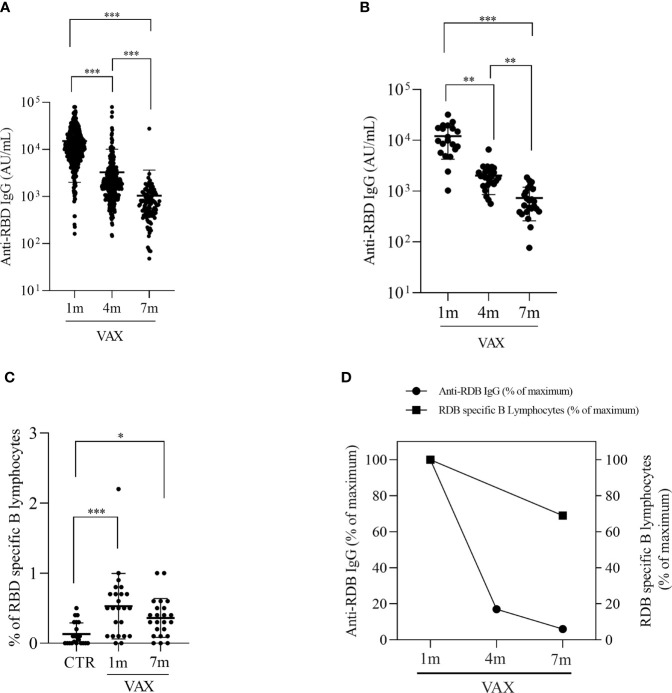
Analysis of anti RBD specific IgG levels and RBD specific B lymphocytes in immunized subjects. **(A)** Concentration of anti-RBD IgG at 1, 4 and 7 months post second dose vaccination in the HCW cohort. Data were were analyzed by repeated-measurement two-way ANOVA and Bonferroni’s multiple comparison test. A two-sided p < 0.05 was considered statistically significant. **(B)** Concentrationof anti-RBD IgG at 1-, 4- and 7- months post vaccination in the subgroup of subjects that were analyzed also for cellular populations. Differences were analyzed with Friedman test and Dunn’s multiple comparison test. **(C)** comparision of circulating RBD specific B lymphocytes in not immunized controls (CTR) and in vaccinated subjects at 1 and 7 months, measured by flow cytometry. **(D)** comparision between the decline in anti-RBD IgG levels and in RBD specific B lymphocytes. Results are expressed as percentage of initial measured levels. *p < 0.05 **p < 0.01. ***p < 0.001.

We enrolled 432 HCW operating at IRCCS San Raffaele Roma to evaluate the long-term efficacy of anti-COVID-19 vaccination, by measuring serum specific antibody levels. The majority of subjects were females (n=250, 57.8% of total; mean age 50, range 27-67), while males accounted for the 42.2% of the cohort (n=182, mean age 51, range 31-72). All the subject that received the vaccine had no previous history of COVID-19. We measured anti SARS-COV2 IgG levels at month 1, 4 and 7 after vaccination. As shown in **Figure 1A**, a dramatic decrease in IgG serum concentration was observed between one and four months after second dose administration (from 15.124,7AU/ml ± 636,5 to 3.260,2AU/ml ± 378,2, 78% reduction, p<0.001). An additional 67.9% decrease was observed between 4 and 7 months (3.260,2AU/ml ± 378,2 to 1.046,6AU/ml ± 248,7 p<0.001), determining an overall 93.1% reduction after 7 months from vaccination. In our cohort of vaccinated individuals, we registered 7 cases of SARS-COV2 infections, which accounts for the 1,6% of total immunized population, indicating a high efficacy of the vaccine in preventing COVID-19 in 7 months follow up. To assess the induction of a spike-specific cellular immunity, we performed flow cytometric analysis of peripheral blood B and T lymphocyte population in a subgroup of 28 vaccinated volunteers at 1 and 7 months after vaccination. These latter results were compared with a control group of 25 subjects who were not yet vaccinated.

Consistently with the general population, also in the subgroup of 28 volunteers, we observed a progressive decline in RBD-specific IgG serum concentration, with a 83.8% reduction at month 4 (12086.45AU/ml ± 1752.44 at month 1 versus 1959.81 AU/ml ± 226.52 at month 4, p=0.014) and a less pronounced decrease, i.e., 45.2% measured at month 7 (month 4: 1959.81AU/ml ± 226.52, month 7: 709.76AU/ml ± 94.68, p=0.014), for a total of 94.1% decrease after vaccination (**Figure 1B**). We analyzed the presence of RBD specific B lymphocytes by incubating cells with a biotynilated RBD, followed by staining with PE-streptavidin. **Figure 1C** shows that a population of spike specific B lymphocytes can be found up to 7 months after vaccination. Gating strategy for RBD-specific B cells identification is reported in **Figure S1**. Spike specific B cells were found to be 0.50% ± 0.13 and 0.38% ± 0.07 of total CD19+ B lymphocytes at month 1 and month 7, respectively, compared with 0.13% ± 0.03 observed in not-immunized individuals (p<0.001). Interestingly, though a decrease in the percentage of RBD-specific B lymphocytes can be observed at 7 months, this difference with the observed value at 1 month is not significant (p=0.732) but the percentage of RBD-specific B lymphocytes in immunized subjects is still higher when compared to not-vaccinated controls (p<0.001), thus suggesting that vaccination is capable to induce a long-lasting defense system ready to respond to a subsequent re-encounter with the antigen. Of note, even if in some subjects we couldn’t identify Spike-specific B lymphocytes, in the majority of samples (20 out of 23, 86%) we observed the presence of RBD-binding B cells. Comparison between the decrease in humoral immunity vs the decrease specific B cellular immunity demonstrate a slower decrease in cellular response (**Figure 1D**).

**Figure 2:**
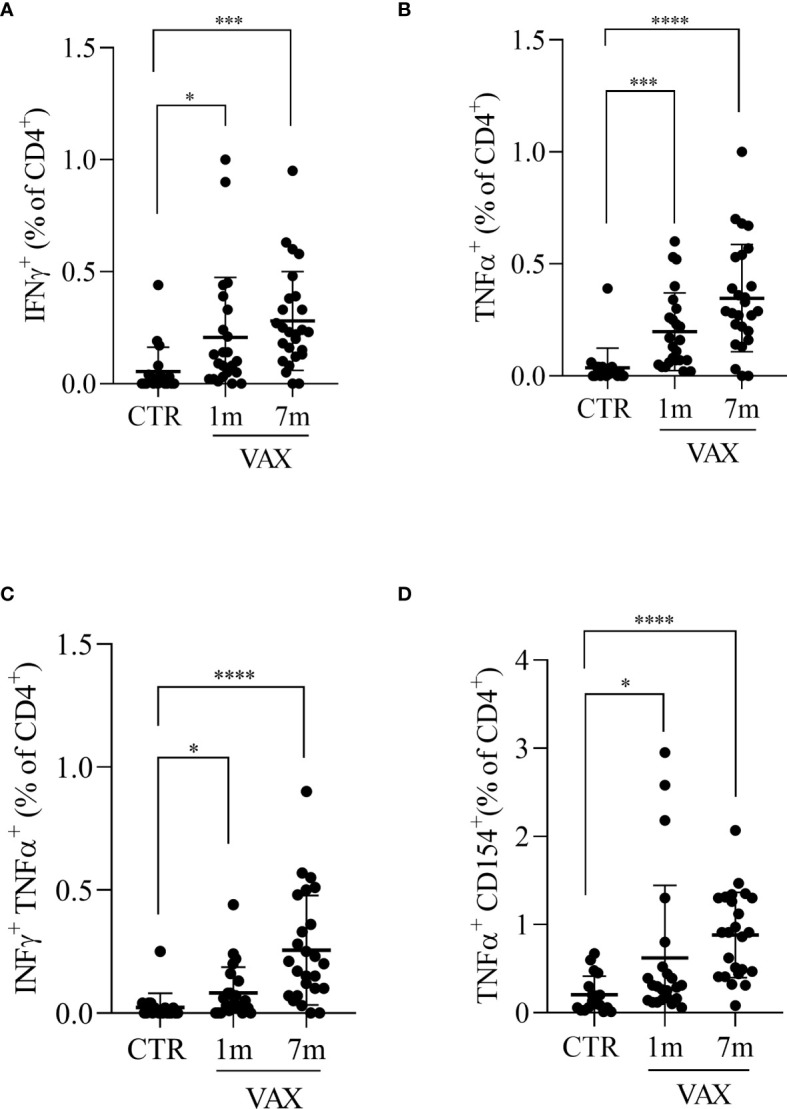
Analysis of CD4 + T lymphocytes response to Spike derived peptides. Total PBMCs were stimulated *in vitro* with a pool of peptide derived from Spike (Wuhan variant). After 6 hours incubation in the presence of brefeldin A during the last 4 hours, PBMCs were stained and analyzed by flow cytometry for the production of INFγ **(A)**, TNFα **(B)**, for the simultaneous production of INFγ and TNFα **(C)**, or for the production of TNFα in CD154+ CD4+ T lymphocytes **(D)**. Cells from immunized subjects, analyzed at 1 and 7 months after vaccination cycle completion, were compared with cells from not-vaccinated controls (CTR). Data were analyzed using Mann-Whitney U-test *p<0.05; ***p< 0.001; ****p<0.0001.

**Figure 3:**
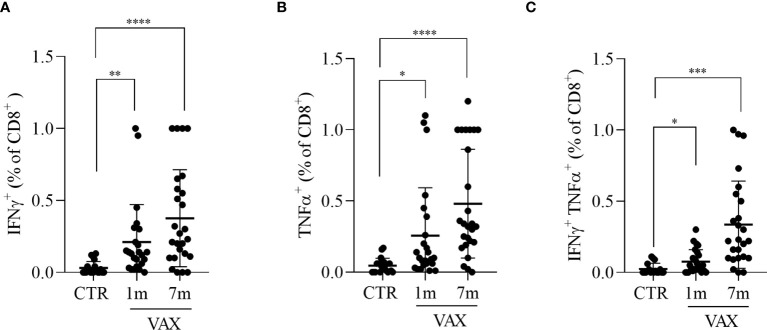
Analysis of CD8+ T lymphocytes response to Spike derived peptides. Total PBMCs were stimulated *in vitro* with a pool of peptide derived from Spike (Wuhan variant). After 6 hours incubation in the presence of brefeldin A during the last 4 hours, PBMCs were stained and analyzed by flow cytometry for the production of INFγ **(A)**, TNFα **(B)**, or for the simultaneous production of INFγ and TNFα **(C)** in CD8+ T cells. Cells from immunized subjects, analyzed at 1 and 7 months after vaccination cycle completion, were compared with cells from not-vaccinated controls (CTR). Data were analyzed using Mann-Whitney U-test *p<0.05; **p<0.01; ***p< 0.001; ****p<0.0001.

We next analyzed the presence of Spike specific T lymphocytes by stimulating *in vitro* PBMC with a pool of peptides derived from Spike, and assessed T cells activation by flow cytometry. To stimulate cells we used peptides derived from the Spike protein of the “wild type” Wuhan variant of the virus. The production of INFγ and TNFα from CD8+ T cells and the production of TNFα, IFNα and the upregulation of CD154 (CD40L) on CD4+ T cells were considered indicative of antigen specific T cell activation. After paired background subtraction from parallel unstimulated cultures, we found that, both at 1 month and 7 months after vaccination, spike-derived peptides induced a specific T cell response, both in CD4+ and in CD8+ T lymphocytes. Gating strategy is depicted in the **Figure S2**. **Figure 2** shows the percentages of Spike-specific CD4 T cells observed in not-immunized subjects (CTR) and in vaccinated individuals at 1 and 7 months. We observed in immunized subjects a higher percentage of Spike-specific CD4 T lymphocytes both at 1 and 7 months, compared to not-vaccinated controls, in all the analyzed populations: INFγ+ CD4+ T cells (0.21 ± 0.05 and 0.28 ± 0.04 versus 0.04 ± 0.02, p<0.05, **Figure 2A**); TNFα+ CD4+ T cells (0.20 ± 0.03 and 0.35 ± 0.05 versus 0.04 ± 0.02, p<0.001, **Figure 2B**); INFγ+ TNFα+ CD4+ T cells (0.08 ± 0.02 and 0.25 ± 0.04 versus 0.02 ± 0.01, p<0.05, **Figure 2C**); and CD154+ TNFα+ CD4 T cells (0.62 ± 0.17 and 0.88 ± 0.10 versus 0.20 ± 0.05, p<0.05, **Figure 2D**). The same analysis was conducted on Spike-specific cytokines producing CD8+ T cells. Accordingly, we found that immunized subjects showed higher percentage of cytokine producing CD8+ T lymphocytes after stimulation with Spike derived peptides, both at 1 month and 7 months after vaccination, when compared to not immunized subjects. The observed percentages of cytokines producing cells in vaccinated versus not immunized workers are shown in **Figure 3**, in particular: INFγ+ CD8+ T (0.21 ± 0.05 at month 1 and 0.37 ± 0.06 at month 7 versus 0.03 ± 0.01 in the controls, p<0.001, **Figure 3A**); TNFα+ CD8+ T (0.25 ± 0.07 at month 1 and 0.48 ± 0.07 at month 7 versus 0.05 ± 0.01 in the controls, p<0.01, **Figure 3B**); and INFγ+ TNFα+ CD8+ T (0.08 ± 0.01 at month 1 and 0.33 ± 0.06 at month 7 versus 0.02 ± 0.01 in the controls, p<0.05, **Figure 3C**). A specific T cell response to spike stimulation was observed in 83% and 79% of subjects at 7 months for CD4+ and CD8+ lymphocytes, respectively.

## Discussion

Vaccine efficacy and persistence of protection in time are a matter of debate both in the scientific community and in the general public. Here, we report the results observed 7 months after completion of vaccination cycle with the BNT162b2 mRNA vaccine in a cohort of health care workers. We first evaluate the serum concentration of anti-RBD specific IG, and found a dramatic decrease in time. However, decrease of specific antibody levels in serum is a common and expected feature of vaccination (11), and should not rise concern as long as the vaccination is able to induce the expansion of B lymphocytes populations specific to the antigen of interest, such as plasma cells that reside in secondary lymphoid tissues and secrete circulating antibodies and memory B cells that enhance protection by secreting antibodies in a paracrine way after pathogen entry (12). Previous reports indicate that vaccination with BNT162b2 vaccine induces the expansion of a population of spike-specific memory B lymphocytes that were still detectable after 3 months post second dose (13). Our results show that 7 months after primary vaccination a population of RBD-binding B lymphocytes, potentially able to steadily secrete specific anti-spike antibodies, can be found in immunized subjects.

Along with B lymphocytes, T lymphocytes are essential players in the protection against viral infection mainly by killing virus-infected host cells (14) and by providing help to B cells in differentiating into memory B cells and in producing antibodies (15). T lymphocytes play a key role in the host defense against SARS-COV2, as T lymphopenia has been correlated to poor prognosis (16). Thus, we have investigated whether *in vitro* stimulation of PBMCs from vaccinated individuals with a pool of peptides derived from the Spike protein resulted in activation of specific CD4+ and CD8+ T lymphocytes, as it was described in COVID-19 patients (17). Our results indicate that mRNA vaccination induces a robust response from cytokines-producing T lymphocytes, that lasts up to 7 months, in keeping with previous results (18, 19) as it is observed also in individuals recovered from SARS-COV2 infection (20, 21). In our *in vitro* experiments, we observed that both CD4+ and CD8+ T lymphocytes were capable to produce IFNg, with small difference between the two subsets. Previous results showed a greater difference in the amount of IFNg produced by CD4+ and CD8+ T cells (22, 23). This apparent discrepancy can be explained considering the different assays used (flow cytometry identification of IFNγ positive cells versus ELISA or ELISPOT assays), but also the subjects included in the studies (immunized subjects or infected patients). We cannot exclude that a quantitative assay such as ELISA performed on our samples could have highlighted the same differences described from other groups. We observed a contraction of the humoral immune response that was evident 4 months after completion of vaccination cycle. However, this contraction is expected after vaccination (9) and it is also observed in follow up studies of COVID-19 recovered patients (24–26), but since no vaccination from Jenner’s first observations to now has been so deeply in the spotlight of media and of public opinion, this gave rise to concerns on the duration and the efficacy of the protection induced by anti-SARS-COV2 vaccination. Our data could slightly put the worries in perspective. Analysis of the immune response to SARS-COV2 infection in patients showed that the severity of the illness was strongly correlated to a decrease in the absolute numbers of T lymphocytes (27), together with a decrease in NK cells numbers (28). Moreover, patients with impaired humoral immunity but conserved T cell populations respond efficiently to SARS-COV2 infection. In addition, in patients who experienced severe COVID-19 T lymphocytes subpopulation were shown to be impaired, with a reduction in the percentages of effector memory CD4+ T helper cells (29), a decrease in the percentage of IFNγ producing cells (30) and in Th17 cells (29) together with an expansion of Th2 lymphocytes (29). Our data shows that mRNA vaccination can induce the expansion of populations of Spike-specific B and T lymphocytes population that persist in time and, in some individuals, appears to be increased at 7 months after vaccination compared to values observed at 1 month. Although we have not analyzed the expression of memory markers, we could assume that the spike specific T lymphocytes producing lymphocytes that we observed, possibly are memory or effect memory cells, given the short stimulation time, as memory cells display a faster production of cytokines, comparing to naïve T cells (31). This would be in keeping with results from Guerrera et al. (19) that observed the prevalence of central memory (CM) and effector memory (EM) subsets in Spike-specific CD4+ T cells and of CM, EM and terminally differentiated (EMRA) subsets in CD8+ T. Specific cellular immunity, mediated by T and B lymphocytes, can steadily respond and restrict viral infection, even in subjects with very low concentration of neutralizing antibodies (32, 33), thus preventing or reducing symptoms of COVID-19, and possibly reducing spreading of virus to others (34, 35).

Our work has strengths and limits. One of the limits is the low number of subjects included in the study on cellular response to Spike, a number that was chosen after calculation of the sample size needed to achieve statistical significance, on the basis of studies performed in COVID patients (17). Another limit is the use of total anti-RBD IgG, instead of neutralizing antibodies, as an indicator of humoral response. Although we are aware that the measurement of neutralizing antibodies gives a better indication of the real effectiveness in blocking the viral infection, we chose to compare the Spike-specific circulating B lymphocytes with the levels of total IgG as this is what diagnostic laboratories measure when people check for their immune status. Moreover, while we analyze the expression of Th1 cytokines (IFNγ and TNFα), we did not investigate whether mRNA vaccination against SARS COV2 is able to induce also a population of Th2 lymphocytes. While there are several reports, direct or indirect (30, 36), of an induction of Th2 lymphocytes in COVID 19 patients, that is associated with a more severe disease and a poorer prognosis, to our knowledge there are no reports that analyzed the presence of Th2 cells induction by mRNA vaccination. The strength of this work is to have analyzed both B and T lymphocytes populations, in order to achieve a broader description of the adaptive immune response.

Our data, together with results from other groups (13, 19) sustain the evidence that immunization induced by mRNA vaccination is efficient and long lasting, even if the decrease in antibody levels fuels the fear of losing protection in time. The emergence of various variants of concern prompted new discussions on the efficacy of currently available vaccines. At the moment, the Delta and the Omicron variants represent the most prevalent variants. Recent evidences, however, demonstrated that T cell response induced by mRNA vaccination is able to recognize both Omicron and Delta, with an efficacy comparable or only slightly reduced compared to the response elicited by the wild type (Whuan) variant (37, 38). In the light of these data, we can assume that the prolonged response that we and others observed in immunized subject is potentially capable to control the infection also from delta and Omicron variants. It is true that some categories of individuals may have a worse response to vaccination (immunocompromised subjects, elderly people) and also in our cohort we could not observe the presence of RBD –specific B lymphocytes in 14% of subjects and lack of T cell specific response in 17% (for CD4+ subset) and 21% (for CD8+ lymphocytes) of vaccinated individuals, however the majority of vaccinated subjects in our observation showed the presence of specific T and B cells population that potentially can protect from SARS-COV2 infection. The presence of non-responding individuals should be kept in mind when considering to reduce containment measures. However, our data show that vaccine is highly effective in inducing a specific cellular response that lasts in months. In light of these results, the need for subsequent booster doses of vaccine could be reconsidered, and priority should be given to those who still didn’t receive even a single dose of vaccine, or to specific fragile populations. To date, there aren’t studies demonstrating a reduced effect of vaccination against severe disease in healthy subjects (39, 40); furthermore, spreading the message that booster doses should be given broadly, and not to selected categories of subjects for which there is a demonstrated need for a subsequent dose, could further reduce confidence in vaccines in those who are already skeptical about their efficacy (41). The currently available vaccines are safe, efficient in preventing the diseases and save lives: priority should be given to those who still haven’t received the primary vaccination and to fragile or highly exposed to risk population, keeping in mind that we are all connected and none of us could be out of risk until we all will be protected. Booster doses would ensure additional protection to already immunized subjects, thus strengthening the individual, but since our aim is to ensure global protection to the whole population we should give priority to primary vaccination.

## Data Availability Statement

The raw data supporting the conclusions of this article will be made available by the authors, without undue reservation.

## Ethics Statement

The studies involving human participants were reviewed and approved by Ethic Committee of IRCCS San Raffaele Roma. The patients/participants provided their written informed consent to participate in this study.

## Author Contributions

Designing research studies, LV, DL, CT, and MF. Conducting experiments, LV. Acquiring data, LV, FC, and MC. Analyzing data, LV, LG, SI, SP, and SB. Writing the manuscript, LV, SI, SB, and PR. All authors contributed to the article and approved the submitted version.

## Funding

The work was supported by Italian Ministry of Health (“Ricerca Corrente”) to IRCCS San Raffaele Roma.

## Conflict of Interest

The authors declare that the research was conducted in the absence of any commercial or financial relationships that could be construed as a potential conflict of interest.

## Publisher’s Note

All claims expressed in this article are solely those of the authors and do not necessarily represent those of their affiliated organizations, or those of the publisher, the editors and the reviewers. Any product that may be evaluated in this article, or claim that may be made by its manufacturer, is not guaranteed or endorsed by the publisher.
