# Supplementary material for: Long Lasting Cellular Immune Response Induced by mRNA Vaccination: Implication for Prevention Strategies
Source: Front Immunol. 2022 Mar 10;13:836495. doi: 10.3389/fimmu.2022.836495 (PMC8961295; doi:10.3389/fimmu.2022.836495)
Supplement: Supplementary file 1 [file Image_1.pdf]

Supplementary Figure

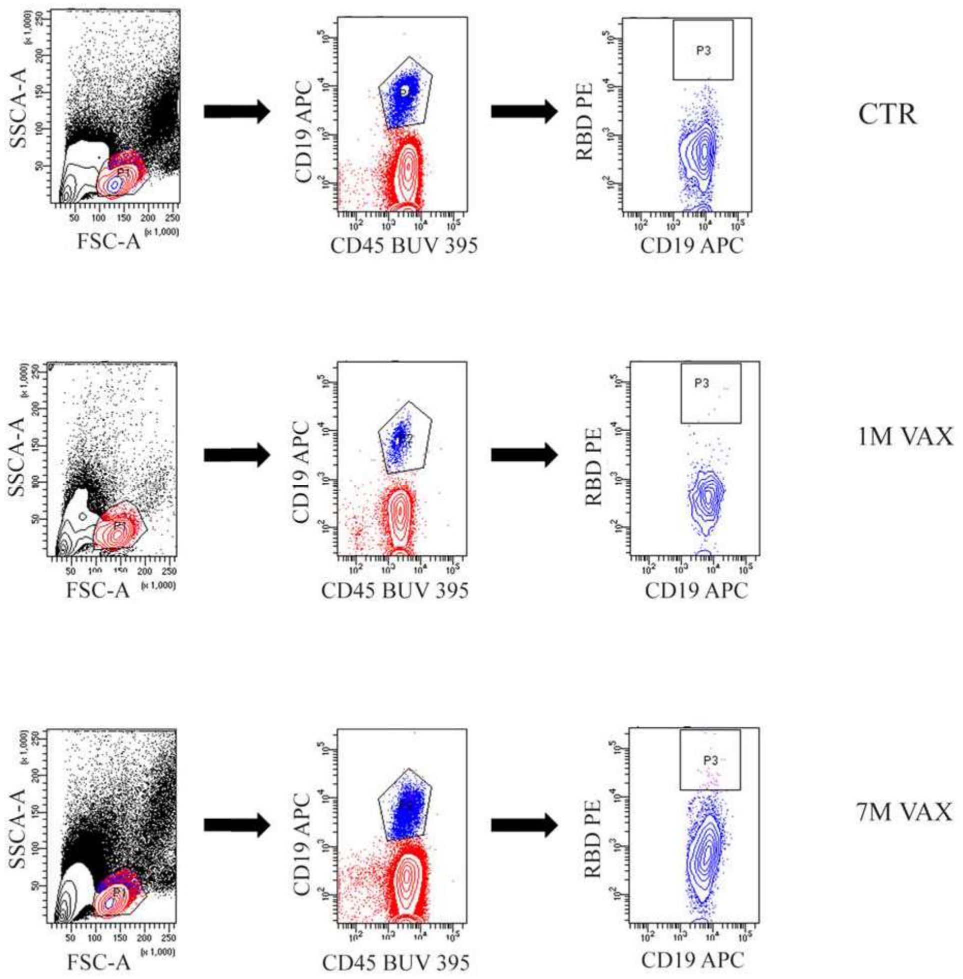

Figure S1. Gating strategy for identification of RBD-binding B lymphocytes. The plots show three representative staining for B lymphocytes in a not immunized subject (upper plots), and in a vaccinated subject 1 months (central plots) and 7 months (lower plots) after vaccination.
