# Supplementary material for: Long Lasting Cellular Immune Response Induced by mRNA Vaccination: Implication for Prevention Strategies
Source: Front Immunol. 2022 Mar 10;13:836495. doi: 10.3389/fimmu.2022.836495 (PMC8961295; doi:10.3389/fimmu.2022.836495)
Supplement: Supplementary file 2 [file Image_2.pdf]

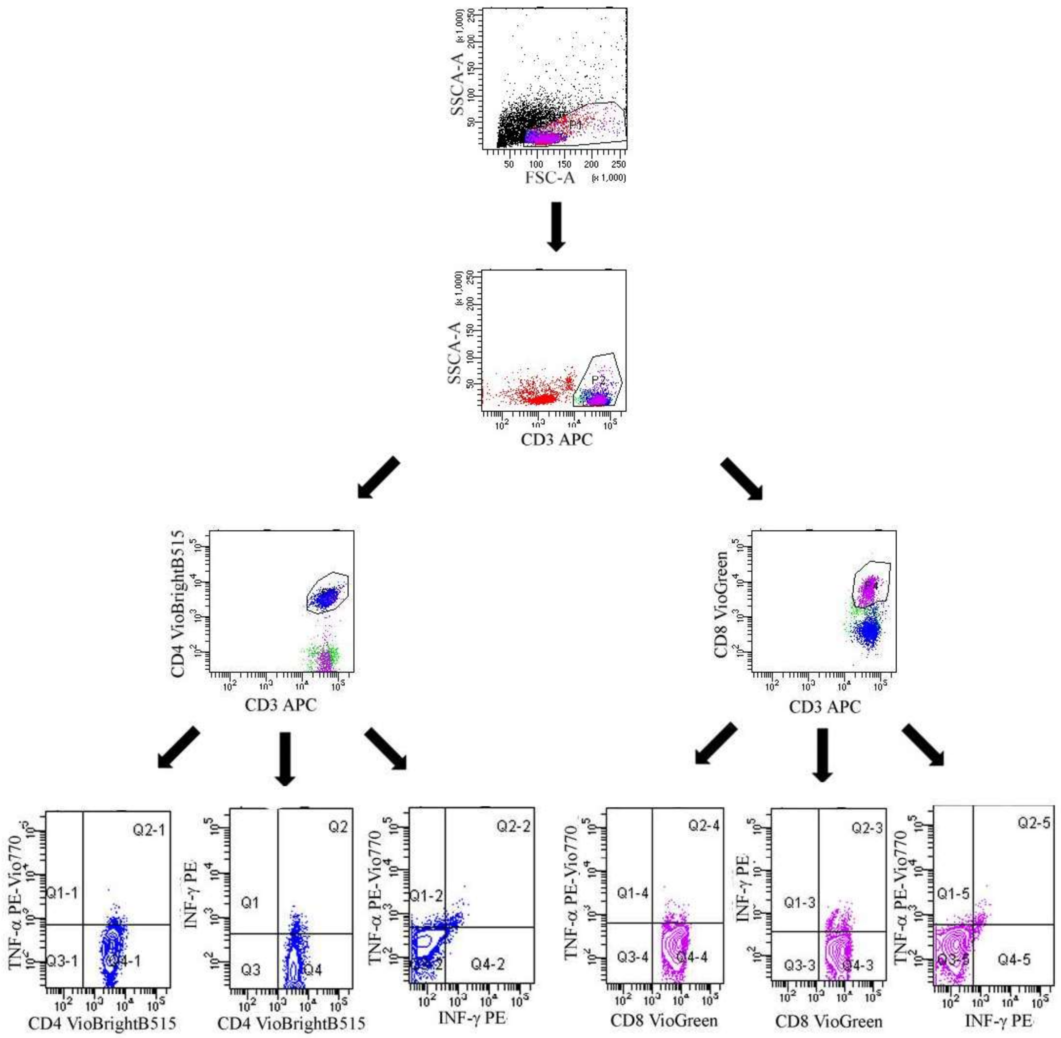

Figure S2. Gating strategy for CD4+ and CD8+ T lymphocytes activated in vitro by Spike derived peptide pools.

The plots show a representative staining for CD4+ and CD8+ T lymphocytes in a vaccinated subject, after 6 hours incubation (in the presence of Brefeldin A for the last 4 hours) with a pools of peptides derived from Sars-Cov2 Spike protein. Labels on the axes indicates the conjugated antibodies used for the staining. The upper right quadrant in the bottom plots contains the cells considered double positive and specifically induced by Spike stimulation (quadrants were defined on unstimulated cells, cultured in parallel).
